# The Thalamocortical Mechanism Underlying the Generation and Regulation of the Auditory Steady-State Responses in Awake Mice

**DOI:** 10.1523/JNEUROSCI.1166-23.2023

**Published:** 2024-01-03

**Authors:** Jinhong Li, Zijie Li, Xueru Wang, Yunhan Liu, Shuai Wang, Xuejiao Wang, Yingna Li, Ling Qin

**Affiliations:** Department of Physiology, China Medical University, Shenyang 110122, People’s Republic of China

**Keywords:** AC, ASSR, gamma oscillation, MGB, sound discrimination, TRN

## Abstract

The auditory steady-state response (ASSR) is a cortical oscillation induced by trains of 40 Hz acoustic stimuli. While the ASSR has been widely used in clinic measurement, the underlying neural mechanism remains poorly understood. In this study, we investigated the contribution of different stages of auditory thalamocortical pathway—medial geniculate body (MGB), thalamic reticular nucleus (TRN), and auditory cortex (AC)—to the generation and regulation of 40 Hz ASSR in C57BL/6 mice of both sexes. We found that the neural response synchronizing to 40 Hz sound stimuli was most prominent in the GABAergic neurons in the granular layer of AC and the ventral division of MGB (MGBv), which were regulated by optogenetic manipulation of TRN neurons. Behavioral experiments confirmed that disrupting TRN activity has a detrimental effect on the ability of mice to discriminate 40 Hz sounds. These findings revealed a thalamocortical mechanism helpful to interpret the results of clinical ASSR examinations.

**Significance Statement** Our study contributes to clarifying the thalamocortical mechanisms underlying the generation and regulation of the auditory steady-state response (ASSR), which is commonly used in both clinical and neuroscience research to assess the integrity of auditory function. Combining a series of electrophysiological and optogenetic experiments, we demonstrate that the generation of cortical ASSR is dependent on the lemniscal thalamocortical projections originating from the ventral division of medial geniculate body to the GABAergic interneurons in the granule layer of the auditory cortex. Furthermore, the thalamocortical process for ASSR is strictly regulated by the activity of thalamic reticular nucleus (TRN) neurons. Behavioral experiments confirmed that dysfunction of TRN would cause a disruption of mice's behavioral performance in the auditory discrimination task.

## Introduction

Auditory steady-state response (ASSR) is a cortical oscillation in the gamma range (30–80 Hz) induced by a sequence of repetitive acoustic stimuli, which can reflect the integrity of the auditory pathways and the capacity of these pathways to generate synchronous activity (Sugiyama et al., 2021). ASSR is one of the event-related potential measures most extensively studied in both clinic and neuroscience research. Electroencephalogram (EEG) and magnetoencephalography (MEG) examinations have revealed that the power and phase locking of ASSR are reduced in patients with neuropsychiatric disorders, such as schizophrenia (SZ; Kwon et al., 1999; O'Donnell et al., 2013; Thune et al., 2016). Furthermore, similar changes are seen in subjects who are at high risk of developing this disease, such as first-degree relatives (Hong et al., 2004). ASSR abnormalities are suggested to be functionally important markers of impaired information processing (Spencer et al., 2008). However, one critical question involving ASSR concerns the underlying neural circuits, which have been studied to only a limited degree.

Most of previous works on ASSR have focused on cortical mechanisms. By simultaneous recording from the electrodes directly implanted in multiple brain areas of rats, we have found the evidence supporting the auditory cortex (AC) as the region of ASSR originator (Y. Wang et al., 2018). Some studies on humans also have localized the source of ASSR at the AC (Herdman et al., 2002, 2003; Kuriki et al., 2013; Klados and Bamidis, 2016). We further analyzed the laminar multielectrode data recorded in the AC and found the ASSR is the strongest in the granular cortical layers (Li et al., 2021). Because the granular layer is the thalamorecipient stage of AC, it is reasonable to consider that the medial geniculate body (MGB) is a lower level contributor for ASSR generation, which provides the dominant afferent inputs for acoustic perception from the thalamus to the AC (Lee, 2013). Besides the sensory thalamocortical pathway, some subcortical modulatory nuclei were also reported to play a regulatory role in the ASSR (Kaas and Hackett, 1998; Reyes et al., 2004; Steinmann and Gutschalk, 2011). The thalamic reticular nucleus (TRN) is a shell-shaped nucleus, which is strategically located between the thalamus and the cortex (Guillery and Harting, 2003). TRN, composed of GABAergic neurons, represents an ideal hub for corticothalamic communications, because it receives excitatory projections from thalamocortical and corticothalamic neurons and sends inhibitory efferents to the thalamus (Y. Li et al., 2020). There is a growing body of evidence from both animal and human studies suggesting that TRN may play a key role in the pathophysiology of neuropsychiatric disorders (Court et al., 1999; R. Smith et al., 2001; Cochran et al., 2002; Egerton et al., 2008; Pratt and Morris, 2015; Steullet et al., 2018). Though it has been proposed that gamma oscillations are likely to be regulated by the TRN influence on thalamocortical loops (Pinault, 2004), whether and how TRN regulates the ASSR remains unclear.

Here, we used local field potential (LFP) and single-unit activity (SUA) recordings in mice coupled with selective optogenetic manipulation to examine how activities of the auditory thalamocortical pathway (AC, MGB, and TRN; Read et al., 2002; Hackett, 2011; Lee, 2013; Pickles, 2015) contribute to the generation and regulation of gamma oscillation responses produced by the ASSR paradigm. We further used a Go/No-go behavioral task to examine the effect of TRN activation on the behavioral performance of a mouse to distinguish 40 Hz sound stimuli. Our experiments will reveal the detailed transmission mechanism of ASSRs among the neural circuit of AC, MGB, and TRN and highlight the potential association between the thalamocortical dysfunction and ASSR deficits observed in clinic.

## Materials and Methods

### Experimental design

All experimental procedures were approved by the Animal Ethics Committee of China Medical University (no. KT2018060). C57BL/6 mice were obtained from Vital River Laboratory. VGAT-ChR2-YFP (stock number: 014548) and Gad2-IRES-Cre (stock number: 010802) mice were obtained from The Jackson Laboratory. Experiments were performed in adult mice between 6 and 8 weeks of age weighing between 18 and 25 g. Mice were housed on a 12/12 h day/night cycle to maintain their normal biorhythms and had ad libitum access to food and water. Effort was made to minimize suffering and discomfort of animals and to reduce the number of the animals used. We conducted optogenetic experiments, electrophysiological recordings, and behavioral experiments on wild-type C57BL/6, VGAT-ChR2-YFP, and Gad2-IRES-Cre male mice (see details below).

### Surgical procedures and viral injection

Mice were anesthetized with isoflurane in conjunction with air (3% for induction and 1–2% for maintenance) and fixed in a stereotaxic apparatus with blunt ear bars (#68001, RWD Life Science). After shaving the hair, an incision was made to expose the skull and then the surface of the skull was cleaned with hydrogen peroxide solution (5%) and dried off with air puffer. The skull was positioned such that the lambda and bregma marks were aligned on the anteroposterior and dorsoventral axes. Small craniotomies (∼1 mm in diameter) were drilled on the skull over the location of the TRN [anteroposterior (AP) = −1.06; mediolateral (ML) = ±2.00 mm; and dorsoventral (DV) = −3.2 mm], MGB (AP = −3.28 mm; ML = ±2.0 mm; DV = −3.0 mm), and AC (AP = −2.9 mm; ML = ±4.0 mm; DV = −2.0 mm). In the Gad2-IRES-cre mice, we injected AAV2/9-EF1α-DIO-eNpHR3.0-EYFP-WPRE-hGH into the TRN and AC separately. In C57BL/6 mice, we injected AAV2/9-CaMKIIα-eNpHR3.0-EYFP-WPRE-hGH or AAV2/9-CaMKIIα-ChR2 (E123T/T159C)-mCherry-WPRE-hGH into the MGB. For each injection, 300 nl of viral solution was injected at a slow flow rate of 20 nl/min to prevent potential damage to the surrounding brain tissue (all viruses were injected at 1.5–2.5 × 10^12^ transducing units per ml, obtained from BrainVTA). The micropipette was left in place for 5 min after infusion to avoid virus overflow. Mice were used for electrophysiological recordings >4 weeks post injection of virus. This ensured strong expression of eNpHR3.0-EYFP or ChR2-EYFP in the target brain region.

### Optical fiber implantation

The optical fiber implantation procedure was carried out under anesthesia using a stereotactic frame, as mentioned previously. An optical fiber (200-μm-diameter core and 0.39 numerical aperture; Newdoon Technology) was held by a stereotaxic manipulator and inserted into the brain. The tip of the optical fiber was located slightly on top (∼200 μm dorsoventral) of the targeted region. A custom-designed headpost was attached to the skull with four screws for head fixation. The optical fiber cannula and headpost were fixed with dental cement for later stimulation and recording. After surgery, the mice were allowed to recover for 2–3 weeks before experiments began. Analgesic (carprofen, 5 mg/kg, i.p.) was injected with carprofen immediately after the surgery for consecutive 3 days.

### Electrophysiological recording

After the animals recovered from surgery, they were habituated with the experimental devices in a sound-attenuated recording room. The mice were transported in their home cage to the recording room, where the head was fixed on a custom-made frame through the headpost. The body rested atop a disk, coated with a sound-attenuating polymer (AMSZ16, Zongdiao) that was mounted on a low-friction, silent rotor (2585858, JingKa). A patch optic cable was connected to the optical fiber cannula implanted on the skull. This habituation procedure lasted 30 min per day and was repeated for 3 d. On the fourth day, the mice were briefly anesthetized with isoflurane, and a craniotomy (1.0 × 1.0 mm, mediolateral × rostrocaudal) was performed on top of the TRN, MGB, and AC separately. A small chamber was built around the craniotomy with UV-cured cement. The dura was removed and the craniotomy was protected with saline. After the animal completely recovered from anesthesia, multichannel probe (A1×16-3.8/5 mm-50-177-A16, NeuroNexus) were mounted on a remotely controlled manipulator (MO-10, Narishige) and penetrated into the target brain regions under auditory monitoring of the recorded electrophysiological signals. Reference and ground electrodes were positioned on the interparietal bone. At the end of each recording session, the chamber was filled with ointment (chloramphenicol ointment, 5%) and sealed with UV-cured cement. The chamber was removed and rebuilt under isoflurane anesthesia before each subsequent recording session. During the experiments, we continuously monitored the eyelid and status of the rotating disk to confirm that all recordings were made in the awake condition. At the end of recording session, a small chamber was built around the craniotomy with cement and filled with ointment. The chamber was removed before each subsequent recording session and rebuilt after recording session. Typically, 3–8 recording sessions were performed on each animal over the course of 1–2 weeks.

LFPs and SUAs were recorded in the presence or absence of auditory stimuli. Signals were acquired with a Tucker-Davis System 3 processor (TDT), pre-amplified by a RA16PA, and sampled at 25 kHz by a RZ-2 base station. Spike detection was performed online using threshold crossing and waveform templates. Spike and LFP waveforms were stored on a hard disk and then imported to MATLAB (MathWorks) for the online averaging and monitoring of neural responses to sound stimuli. Offline spike sorting using OpenSorter software (TDT) was performed to include only single-unit spikes in the analysis.

### Optoelectrode recording

Optoelectrodes were custom-made by using the methods described previously (Ono et al., 2018). In brief, we made glass pipettes by pulling borosilicate glass capillaries (Sutter Instrument Company) with an electrode puller (P-97 Flaming/Brown; Sutter Instrument Company). Their tip diameter was 2–3 µm, and their resistance was 4–6 MΩ when they were filled with in 10 mM PBS. The optoelectrode was held by a hollow plastic sleeve that allows for the passage of optical fiber and insulation-coated silver wire (785500, A-M Systems). The silver wire was connected to the headstage of the recording amplifier, and the optical fiber was connected to the laser driver (BT-Aurora-300-470, Newdoon Technology). During the experiment, the optoelectrode was inserted into the granular layer of the AC or TRN under the control of a manipulator (MO-10, Narishige). Once we reached the interest depth, we searched for and isolated the single-unit spikes by adjusting the depth in 2–5 µm steps. After isolating the single unit, we delivered the laser and observed the response to the light stimuli. In some cases, the other electrode held by a manipulator was placed on the surface of the AC to record LFP of the superficial layers of the AC.

### Optogenetic stimulation

Laser stimuli were generated via a laser driver and delivered to the brain through a 200-μm-diameter optical fiber. The intensity of the laser stimulus was measured by an optical power meter (Newdoon Technology) and calibrated to 0.83–3.33 mW mm^−2^ at the tip. A 80 Hz laser stimulus (473 nm, ∼8 mW, 0.5 ms width, 1.0 s total duration) was used to selectively activate the TRN in VGAT-ChR2-YFP mice. A 40 Hz laser stimulus (473 nm, ∼8 mW, 0.5 ms width, 0.5 s total duration) was used to activate the AC. A constant laser stimulus (589 nm, ∼8 mW, 1,000 ms width, 1.0 s total duration) was used to selectively inactivate the TRN and AC.

In one session, 120 trials of a specific pattern of stimulation were presented at a random interval between 4 and 8 s. In the sessions to examine the effect of TRN activation on the ASSR, the laser stimuli were coupled with acoustic stimuli at 250 ms lag of onset. To minimize the rebound effect of neuronal activity, we applied ramping during the 100 ms period at the beginning and end of light stimulation. This approach effectively reduced the abrupt changes in neuronal responses that can occur following sudden changes in input, making the recorded responses more stable and dependable.

### Acoustic stimuli

ASSR was accessed by click-trains, which were a train of rectangular pulse of a 0.2 ms duration, repeated at a rate of 40 pulses/s and continued for 0.5 s duration. The waveforms of sound stimuli were generated digitally with a 100 kHz sampling rate using a custom-built MATLAB (MathWorks) program and transferred to an analog signal by a D/A board (PCI-6052E, National Instruments) and then played through an open-field loudspeaker (K701, AKG) placed at 50 cm away from the ear of the animal opposite to the recording side. The intensity of the sound stimulus was adjusted to be at 50 dB SPL and measured at the center of the recording box (Bruel & Kjær type 2238 sound level meter). We did not use high-intensity stimulus to avoid evoking the startle response in awake mice.

### Go/No-go sound discrimination task

Prior to beginning the training, mice were deprived of water for 24 h. During the training, water was provided only as a reward for correct performance. Sounds for the training were generated by a MATLAB program and delivered through standard computer speakers controlled by an on-board sound card. Speaker calibration was performed prior to the use of the speakers to ensure that the sound intensity remained constant at a level of 50 dB. In the Go/No-go task, a 500 ms sound (40 vs 4 Hz click-train) was played. After a grace period of 1.5 s during which licking had no consequence, mice were rewarded with 2 μl water for licking response to the 40 Hz click-train (hit), while licking to the 4 Hz click-train (false alarm) was met with punishment by means of by 5 s time-out. No licking response to the 40 or 4 Hz click-train was deemed as miss or correct reject, respectively.

During training, 40 and 4 Hz sounds were randomly played to the mice in a 1 : 1 ratio, with 300 trials per day. A typical behavioral session lasted between 1 and 2 h. Mice obtained all of their water in the behavior apparatus (∼1 ml per day; 0.3 ml was supplemented if mice drank <0.5 ml). Mice were then trained daily to the normal task for a period of 2–3 weeks until they displayed stable behavior as measure by their lick probability for all conditions and discrimination performance. Body weight was monitored daily and was kept above 80% of the weight measured prior to water-restriction. Mice received at least 1 ml of water per day, either during behavioral training or in their home cage. After 14 d of water-restriction schedule, mice were given access to water ad libitum for at least 2 consecutive days. The training continued until the mice maintained a performance accuracy of 75% or greater for 5 consecutive days before proceeding to the next experiment.

After completed the training, the mice were assigned a testing session, in which click-trains with five different rates (2, 4, 10, 20, 30, and 40 Hz) were randomly present, and only 40 Hz click-trains were coupled with a reward. Optogenetic activation or inhibition of TRN was randomly added during the sound presentation period in half of the trials.

### Analysis of LFP data

The probe output was delivered to a multichannel preamplifier (RA16PA; TDT) and then to a digital signal processing module (RZ-5; TDT). The signal was amplified and split into LFP (0.1–300 Hz) and SUA (300–5,000 Hz) range by filtering. The current source density (CSD) was calculated as the second spatial derivative of the LFP signal according to the following formula:

$$\mathrm{CSD}=\frac{\left(V_{h\text{-}\Delta h}-2V_{h}+V_{h\;+\Delta h}\right)}{\Delta h^{2}}$$

where *V* is the potential at position h and Δh is the distance between the electrodes. CSD represents the net current density entering or leaving the extracellular medium at a particular spatial position. Columnar CSD patterns evoked by click-train were used to determine the layer of the AC. The granular layer of the AC was determined by a brief current sink appearing at about 10 ms after the sound started (Kaur et al., 2005; Leszczynski et al., 2020). Spectral analysis was conducted on the LFPs using a wavelet-based algorithm, implemented in the function of “timef” in eeglab toolbox (https://sccn.ucsd.edu/eeglab/index.php). The trial-based spectrums of LFPs were accessed by the mean trial power (MTP) analysis (Delorme and Makeig, 2004). MTP was computed by averaging the LFP power in the spectrotemporal domain across the 120 trials from one session. The results of MTP were presented following a dB baseline correction implanted by eeglab.

### Analysis of SUA data

We computed the instantaneous firing rate from each SUA, which was used to construct peristimulus time histograms (PSTHs) and raster plots during the analysis window. PSTH for each neuron and each condition was computed by using the start times of each trial to extract spike times and then convert these to 1 ms bin size histograms. Then, trials from a given condition were averaged together to produce the average PSTH. For each neuron, the mean and standard deviation of the spike counts across trials was computed and then used to produce a *z*-scored PSTH by first subtracting the baseline mean and then dividing by the baseline standard deviation. In this way, we could more easily compare activity of neurons with very different baseline firing rates. For illustration, these normalized PSTHs were smoothed with a 5 ms Gaussian sliding window.

Spike-stimulus phase-locking (SSP) refers to the synchronization between neuronal spiking and the phase of a specific stimulus. Mean vector length (MVL) is a measure that quantifies the degree of SSP, utilizes phase angle and magnitude of each complex number (i.e., each data point) of the corresponding analytic signal in a quite direct way to estimate the degree of coupling. Each complex value of the analytic time series is a vector in the polar plane. Averaging all vectors creates a mean vector with a specific phase and length. The formula is as follows:

$$\mathrm{MVL}=\left|\frac{\sum_{t=1}^{n}\;e^{i\theta_{t}}}{n}\right|$$

where *n* is the total number of data points, *t* is the data point, and $\theta_{t}$ is the phase angle at data point t. This value cannot become negative because it represents the length of the mean vector (Hulsemann et al., 2019).

### Histology

To confirm the position of electrophysiological recording or virus injection, each mouse was anesthetized with pentobarbital (100 mg/kg) after completing all experiments and then perfused transcardially with 30 ml phosphate-buffered saline followed by 4% paraformaldehyde. The brains were removed and placed in a fixative solution for 48 h. Brain samples were sectioned into 20-μm-thick coronal slices via a freezing microtome (Minux FS800, RWD Life Science). Then, brain slices were washed three times in PBS, mounted on glass slides, and coverslipped using DAPI (SL1841, Coolaber). NeuN staining was achieved by antibody incubation with rabbit anti-NeuN polyclonal antibody (1 : 500, Cat# ab177487; Abcam) overnight at 4°C. Markers were visualized with the second antibody of Alexa Fluor 594 conjugate anti-rabbit IgG (1 : 200; Cat# SA00013-4; Proteintech) at room temperature for 1 h.

The recording electrode and virus injection sites were confirmed with the position according to the Paxinos and Franklin mouse brain atlas (3rd edition) under a microscope (BX53, Olympus), fluorescence images of NeuN staining were acquired using a laser scanning confocal microscope (Nikon AXR). Only data from mice in which the AAV infection and the position of the electrodes and optical fiber were correctly located were included for further analysis.

### Statistical analysis

One-way analysis of variance (ANOVA) was performed on the comparisons between the data of different groups. Each ANOVA reporting significant effects was followed by Tukey's post hoc test of multiple comparisons. Paired data were analyzed via paired Student's *t* tests. Statistical significance was determined at *p < 0.05 and **p < 0.01. Data were presented as mean ± standard error (SE).

## Results

### Profiles of neural response in the AC to 40 Hz click-train stimuli

**Figure 1.**
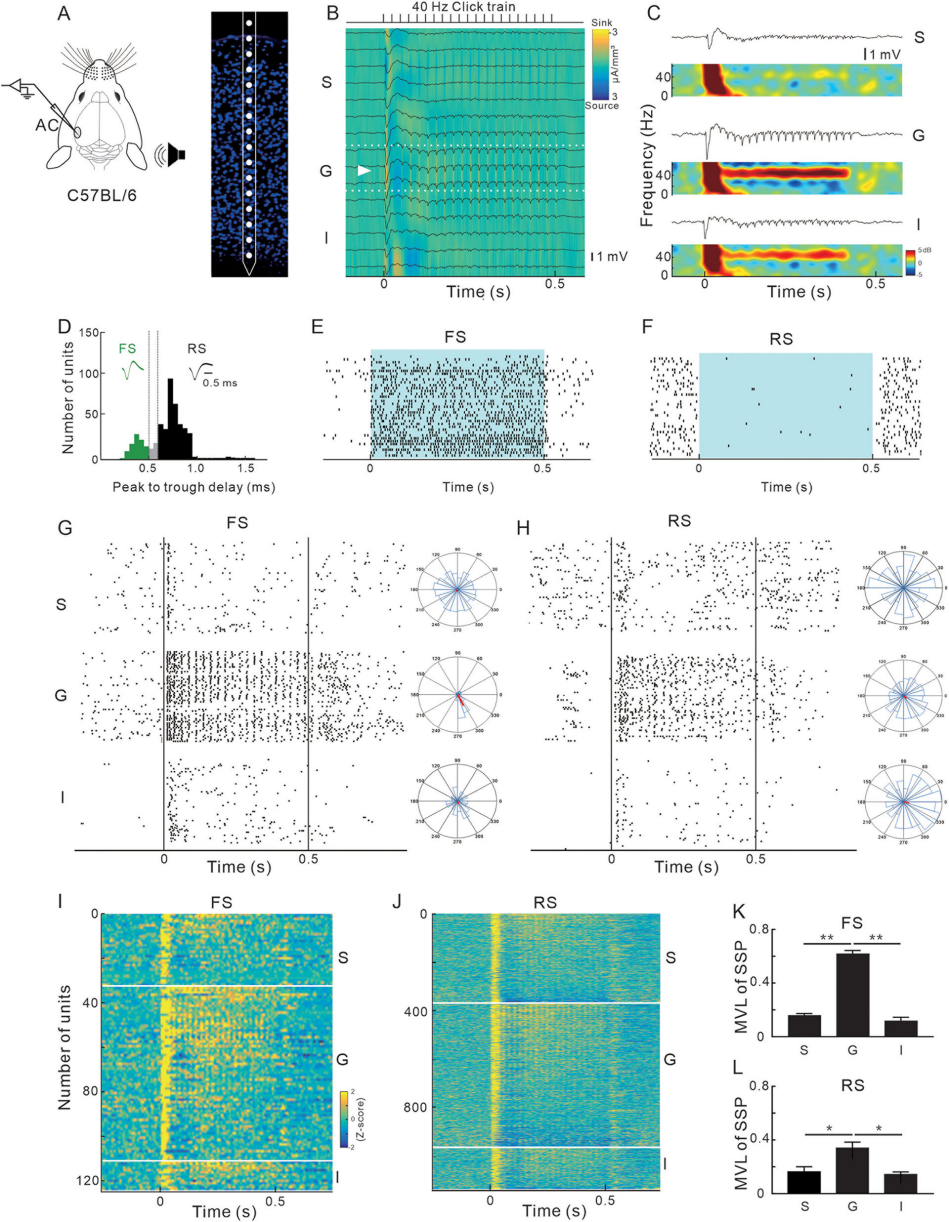
Response profiles of AC neurons evoked by 40 Hz click-train. ***A***, Schematic diagram of columnar recording in the AC by a linear multichannel probe and laminar structure of AC. ***B***, Image of CSD derived from the LFP traces (black). The white triangle indicates a brief, early sound-evoked current sink to identify the G layer. Dotted lines indicate the boundaries of the G layer. ***C***, LFPs simultaneously recorded across different depths of AC and representatives from the S, G, and I groups. Color map presents the result of spectrum analysis of LFP. ***D***, Isolated single units were classified as FS or RS (trough-to-peak delay <0.5 ms or >0.6 ms, dashed vertical lines). Spike waveforms reflect mean FS (green) and RS (black) waveforms. ***E***, ***F***, Raster plot shows the spike time of FS and RS units in response to the blue light stimulation on the AC of VAGT-ChaR2 mouse (shaded area). Firing rate of FS neurons was increased by light stimulation, while that of RS neurons was decreased. ***G***, ***H***, Raster plot shows the 40 Hz click-train-evoked response of representative FS and RS units recorded in the S, G, and I layers, respectively. Vertical lines mark the onset and offset of click-train. Polar diagram is the distribution of spike time relative to the phase of stimulus periodicity; the red bar represents the mean value. ***I***, ***J***, Average *z*-scored responses of all FS and RS units, to 40 Hz click-train stimulation. Each row is one unit, sorted first by cortical layer and second by firing rate (color). All units recorded in AC are included. Unit count within each layer is shown on the left side of each panel. Stimulus period is 0–0.5 s. ***K***, ***L***, Comparison of MVL across different layers (FS, *n* = 126 units; RS, *n* = 1133 units from 23 mice; *p < 0.05; **p < 0.01; ANOVA followed by Tukey's post hoc test).

We firstly investigated the laminar patterns of neural responses to 40 Hz click-train stimuli to reveal laminar profiles of ASSR in the AC. A 1-shank 16-channel silicon electrode probe (A1×16-3.8 mm-50-177, NeuroNexus) was inserted into the AC region to record LFP and SUA (Fig. 1*A*). By utilizing the method of CSD analysis on the LFP signals (Z. Li et al., 2021), we were able to discern the borders that differentiated the layers, specifically, the supragranular (S), granular (G), and infragranular (I) layers (Fig. 1*B*). The average waveform and spectral analysis of LFPs, encompassing the S, G, and I layers, are shown in Figure 1*C*. The results suggest that the G layer exhibited the most robust ASSR, which was synchronized with the 40 Hz click-train stimulus.

SUAs were recorded across AC layers and classified into fast spiking units (FS) and regular spiking units (RS) based on waveform shape identifying putative interneurons and putative pyramidal neurons (Mohan et al., 2019). Figure 1*D* shows the frequency distribution of trough-to-peak delay for the isolated single units. FS and RS were divided by the boundary of 0.5–0.6 ms (dashed vertical lines). In some experiments, we validated the neural classification via optoelectrode methodology to differentiate between the SUAs of GABAergic and non-GABAergic neurons in VGAT-ChR2-EYFP mice (as illustrated in Fig. 1*E,F*). The SUA of a GABAergic neuron was increased during the period of photostimulation, while that of a non-GABAergic neuron was suppressed. The spike waves of GABAergic and non-GABAergic neurons were consistent with those observed between FS and RS, as shown in Figure 1*D*.

We found that RSs and FSs in different AC layers displayed distinct patterns of response to the 40 Hz click-train stimuli. The representative examples of FS and RS recorded in the S, G, and I layers are presented in Figure 1*G*,*H*, respectively. The population results are depicted in the color-scaled PSTHs in Figure 1*I,J*. The FS located in the S layer demonstrated a transient response at both stimulus onset and offset. The FS in the G layer exhibited a rhythmic response that was continuously synchronized to the 40 Hz click-train stimulus. The FS in the I layer showed a sustained response but was not synchronized to the stimulus. The RSs located in the S, G, and I layers demonstrated similar response patterns. However, the synchronizing capability of RSs in the G layer was relatively lower in comparison to the FSs.

The capability of SUA to synchronize with the stimulus was quantitatively evaluated by calculating MVL of SSP (see Materials and Methods). As depicted by the insect polar diagram in Figure 1*G*,*H*, FSs in the G layer mostly occurred at a specific phase relative to the stimulus, with a vector angle between 270° and 300°. The mean MVL of FSs was significantly higher in the G layer compared to that in the S and I layers (Fig. 1*K*; one-way ANOVA; *F*_(2,123)_ = 186.40; *p* < 0.0001; followed by Tukey's multiple comparisons test; *p* < 0.01). The mean MVL of RSs was also higher in the G layer compared to that in the S and I layers (Fig. 1*L*; one-way ANOVA; *F*_(2,1130)_ = 5.377; *p* = 0.0047; followed by Tukey's multiple comparisons test; *p* < 0.05). Taken together, these results suggest that the synchronized spike activities generated by the GABAergic neurons in the G layer contribute most to the 40 Hz ASSR in the AC.

### Profiles of neural response in the MGB and TRN to 40 Hz click-train stimuli

**Figure 2.**
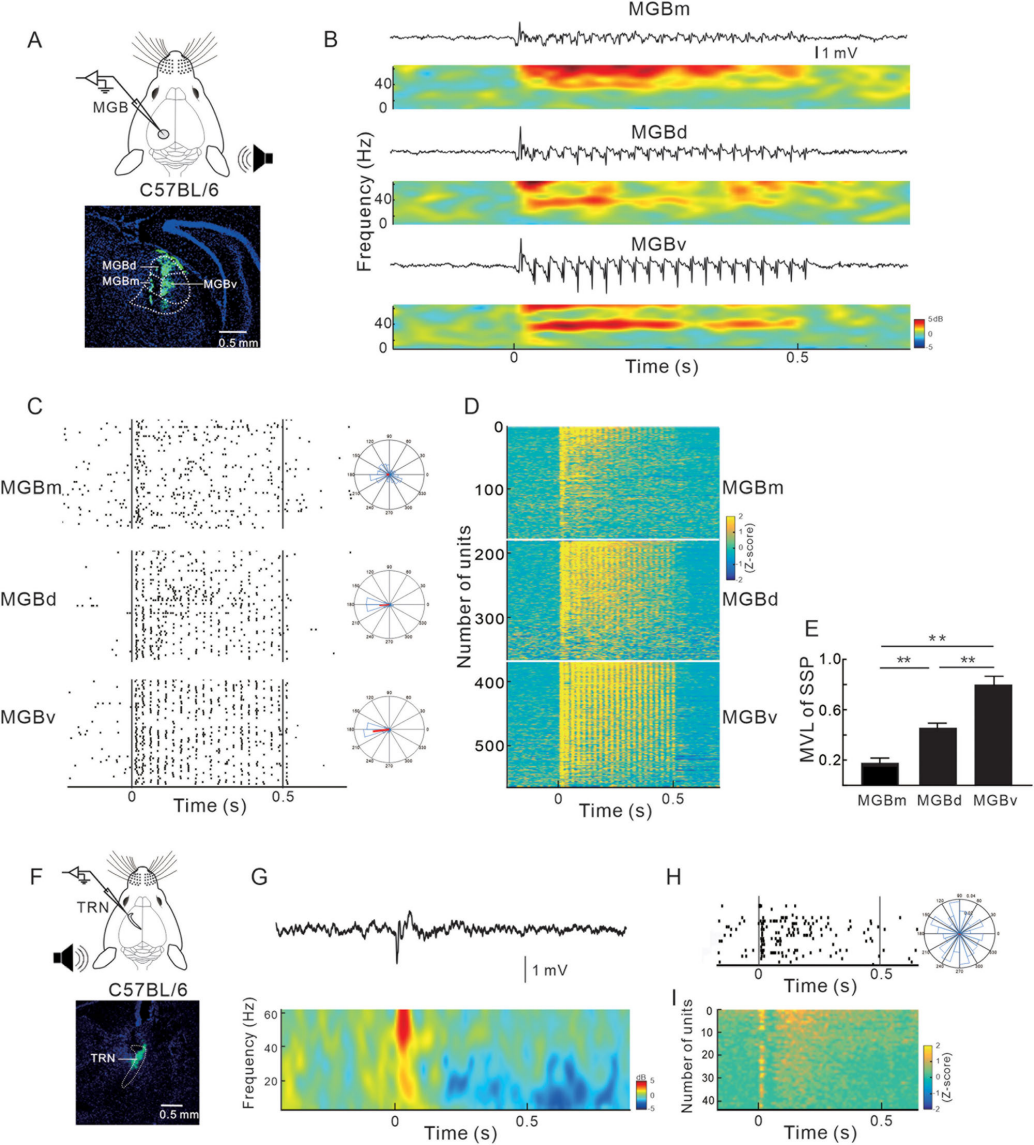
Response profiles of MGB and TRN neurons evoked by 40-Hz click-train. ***A***, Schematic diagram of recording in the MGB and histological confirmation of recording site. ***B***, Representative waveform and spectrum of LFP recorded from the MGBm, MGBd, and MGBv. Stimulus period of 40 Hz click-train is 0–0.5 s. ***C***, Raster plot shows the 40 Hz click-train-evoked response of representative unit recorded in MGBm, MGBd, and MGBv, respectively. Same format as Figure 1*G*. ***D***, Average *z*-scored responses of all MGB units. Same format as Figure 1*I*. ***E***, Comparison of MVL across different divisions of MGB (*n* = 580 units from 12 mice; **p < 0.01; ANOVA followed by Tukey's post hoc test). ***F***, Schematic diagram of recording in the TRN and histological confirmation of recording site. ***G***, Representative waveform and spectrum of LFP recorded from the TRN. ***H***, Raster plot of a representative TRN unit. ***I***, Average *z*-scored responses of all TRN units.

We further recorded the LFPs and SUAs in the MGB in response to 40 Hz click-train (Fig. 2*A–E*). The LFPs in the ventral division of MGB (MGBv) exhibited the most robust 40 Hz ASSR (Fig. 2*B*), when compared to the dorsal and medial division of MGB (MGBd and MGBm). The SUAs in MGBv exhibited consistent synchronization with the 40 Hz click-train stimulus, while the response of SUA in MGBm dominated at the onset of the stimulus (Fig. 2*C*,*D*). The response patterns of MGBd were intermediate between MGBv and MGBm. It is noted that the vector angle of MGBd and MGBv neurons directed at 180°, contrasting to the 270° vector angle of FSs in the G layer of AC. The phase shift between the MGB and AC neurons may reflect a delay of thalamocortical conduction. The mean MVL was the highest in MGBv (Fig. 2*E*) and the MVL difference among the three MGB divisions was statistically significant (one-way ANOVA; *F*_(2,557)_ = 89.24; *p* < 0.0001; followed by Tukey's multiple comparisons test; *p* < 0.01). In light of the fact that MGBv serves as the principal input source of the G layer of the AC (Lee and Winer, 2008; P. Smith et al., 2012), it can be supposed that MGBv may act as the thalamic origin of cortical ASSR. We also recorded the LFPs and SUAs of TRN in response to 40 Hz click-train stimulus (Fig. 2*F–I*). However, no evident 40 Hz ASSR was observed in the LFPs (Fig. 2*G*), and the SUAs displayed a sustained response that was not synchronized to the stimulus (Fig. 2*H,I*).

### Involvement of GABAergic neural activity in the G layer of AC in the generation of 40 Hz ASSR

**Figure 3.**
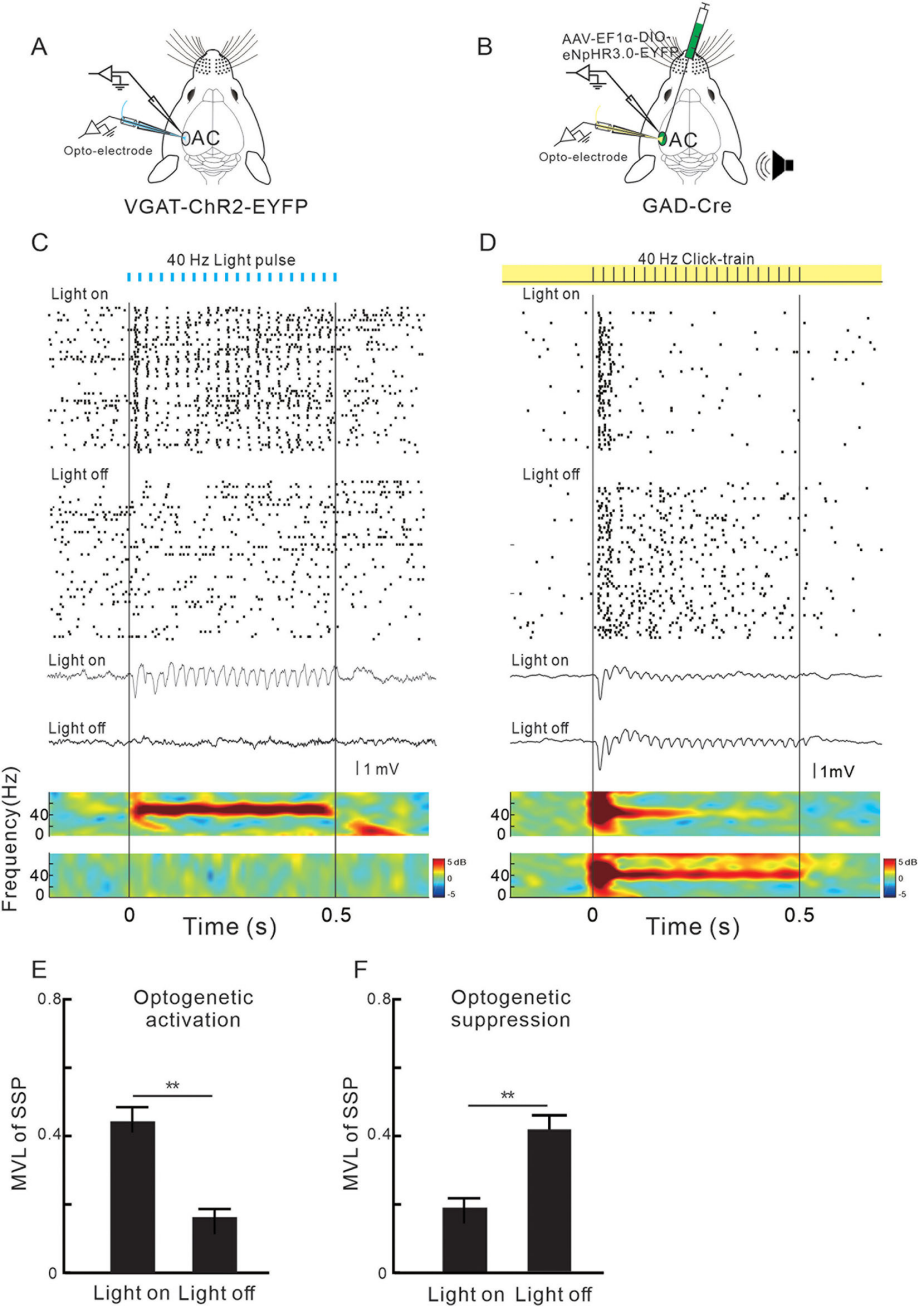
Association between the activity of AC GABAergic neurons and ASSR of LFP. ***A***, ***B***, Schematic diagram of optogenetic manipulation and recording in the AC. ***C***, SUA of a GABAergic neuron and LFP simultaneously recorded in the surface of AC with or without 40 Hz blue light stimulation. Vertical lines mark the onset and offset of photostimulation. ***D***, SUA of a GABAergic neuron and surface LFP in response to 40 Hz sound stimulation with or without continuous yellow light stimulation. ***E***, ***F***, Comparison of MVL between the conditions with and without photostimulation (*n* = 26 units from 5 mice; **p < 0.01; paired Student's *t* tests).

We used an optoelectrode to manipulate the activity of GABAergic neuron in the G layer of AC and simultaneously recorded the LFP from the cortical surface to investigate the contribution of GABAergic neuron in the generation of ASSR (Fig. 3*A*,*B*). A train of light pulses (wavelength of 470 nm) operating at a repetition rate of 40 Hz (Fig. 3*C*) could drive a GABAergic neuron to produce a synchronized response. Additionally, simultaneous recordings of LFP in the cortical surface exhibited a strong and continuous 40 Hz ASSR, as depicted in Figure 3*C*. In contrast, the exposure of yellow light (589 nm) suppressed the synchronized response of GABAergic neurons induced by the 40 Hz click-train sound (Fig. 3*D*). As a result, there was a significant reduction in the 40 Hz ASSR of LFP. The mean MVL was significantly increased or decreased by the optogenetic activation or suppression of GABAergic neurons (Fig. 3*E,F*; *p* < 0.01; paired Student's *t* tests). These results suggest that the activity of GABAergic neurons is crucial for the generation of cortical ASSR.

### Contribution of the MGBv–AC projection to the 40 Hz ASSR in the G layer of AC

**Figure 4.**
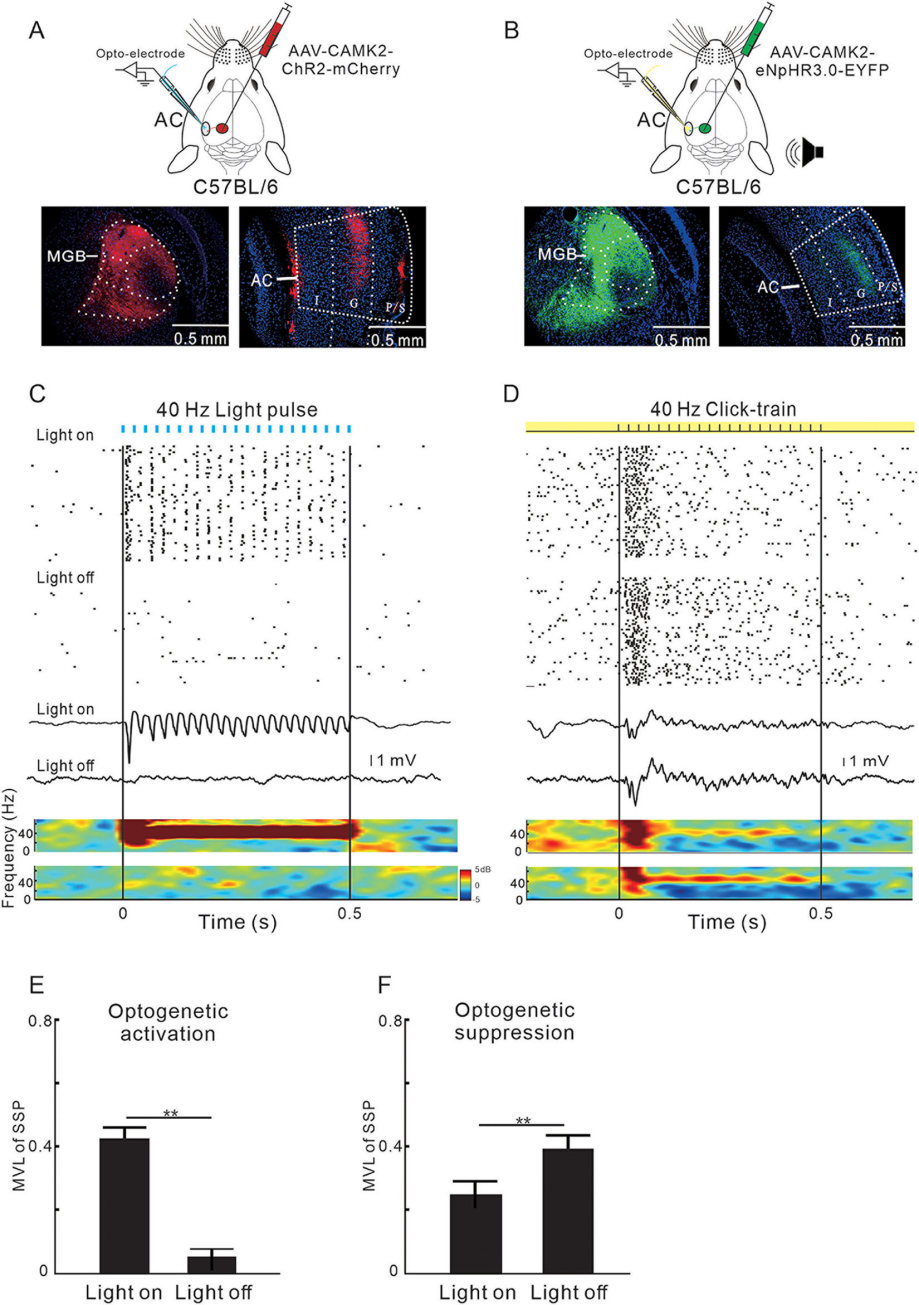
Association between the activity of MGB–AC projection and ASSR of LFP. ***A***, ***B***, Schematic diagram of virus injection in the MGB, optogenetic activation and recording in the AC, histological sections showing the MGB–AC projections. ***C***, SUA and LFP simultaneously recorded in the AC with or without 40 Hz blue light stimulation on the projection from MGB to AC. ***D***, Effects of optogenetic suppression of MGB–AC projection on the SUA and LFP responses evoked by 40 Hz sound stimulation. ***E***, ***F***, Comparison of MVL between the conditions with and without photostimulation (*n* = 33 units from 6 mice; **p < 0.01; paired Student's *t* tests).

We injected AAV-CaMKIIα-ChR2 or NPHR3.0 virus into the MGBv and then used an optoelectrode to investigate the effect of optogenetic manipulation of the MGBv–AC projection on the SUA and LFP in the G layer (Fig. 4). Figure 4*A,B* depict the expression of optogenetic protein in the injection site and their projections in the G layer of AC. High magnification imaging of confocal laser microscope indicates that there was no trans-synaptic spread of virus from the thalamocortical fibers to the AC neurons (Extended Data Fig. 4-1). Stimulation of the MGBv–AC projections with 40 Hz blue light pulses resulted in a synchronized SUA response and 40 Hz ASSR of LFP (Fig. 4*C*). Conversely, suppressing the MGBv–AC projections by continuous yellow light stimulation led to a significant reduction in the synchronized response of SUA and ASSR of LFP that was induced by 40 Hz click-train sound (Fig. 4*D*). The mean MVL between the spike of AC neuron and stimulus was significantly increased or decreased by the optogenetic activation or suppression of MGBv–AC projections (Fig. 4*E,F*; *p* < 0.01; paired Student's *t* tests). Thus, the neural projections from MGBv to the G layer of AC are involved in the generation of ASSR.

10.1523/JNEUROSCI.1166-23.2023.f4-1 Figure 4-1 (A-B) Laser confocal photographs of DAPI/EYFP/NeuN in the G layer of AC. Blue fluorescence represents nuclear staining (DAPI), green fluorescence represents axonal fibers originating from ChR2-EYFP-expressing projection neurons in the MGBv and red represents NeuN staining. The scale bar in (A) represents 100 μm, 20 μm in (D). Download Figure 4-1, TIF file.

### Optogenetic activation of TRN neurons suppressed the 40 Hz ASSR in the MGB and AC

**Figure 5.**
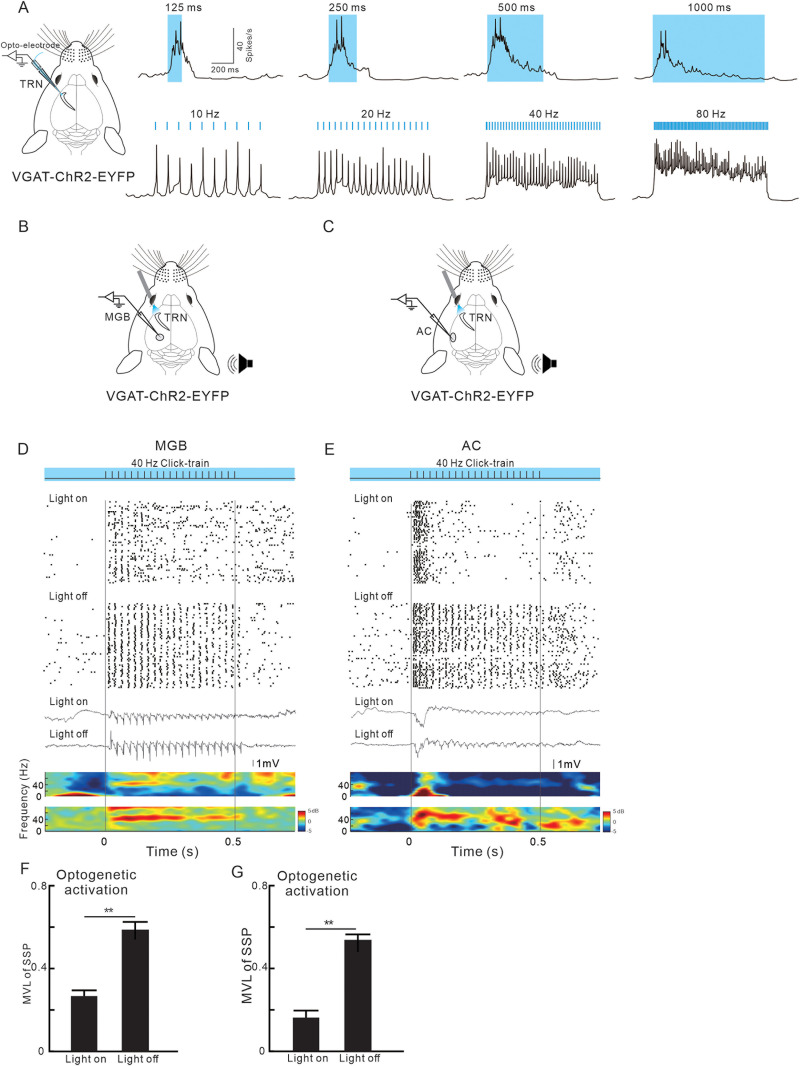
Effect of optogenetic activation of TRN neurons on sound-evoked responses in the MGB and AC. ***A***, Profiles of TRN SUA in response to blue light stimuli with different durations or repetition rates. ***B***, ***C***, Schematic diagram of optogenetic activation of TRN and recording in the MGB and AC, respectively. ***D***, ***E***, SUA and LFP, in the MGB or AC, respectively, in response to 40 Hz sound stimulation with or without blue light stimulation on the TRN. ***F***, ***G***, Comparison of MVL between the conditions with and without photostimulation (*n* = 35 units from 6 mice; **p < 0.01; paired Student's *t* tests).

We further explored how the ASSRs in the MGB and AC are modulated by the activation of the TRN. As illustrated in Figure 5*A*, we utilized an optoelectrode in an in vivo experiment to evaluate the responsiveness of TRN neurons to photostimulation. The results revealed that continuous blue light stimulation lasting over 125 ms effectively induced a pronounced neural discharge in the TRN of VGAT-Chr2-EYFP mice. However, the evoked discharges rapidly adapted as the stimulation duration increased and could not maintain a heightened level for longer than 500 ms. When stimulated by a train of light pulses with a repetition rate of 10 Hz, the TRN neuron exhibited a sequence of discrete discharges that synchronized with the stimuli. As the repetition rate was increased, the evoked discharges gradually fused together, ultimately culminating in a sustained response when the stimulation rate reached 80 Hz. We then used 80 Hz light pulse train to activate TRN and recorded the SUA and LFP in the MGBv and AC in response to 40 Hz click-train sound stimuli (Fig. 5*B,C*). The results, as presented in Figure 5*D*,*E*, revealed that both the SUA and LFP responses, which initially synchronized with the 40 Hz click-train sound, were substantially suppressed by TRN activation. Consequently, the mean MVLs were significantly reduced by the optogenetic activation of TRN (Fig. 5*F*,*G*; *p* < 0.01; paired Student's *t* tests).

### Optogenetic suppression of TRN neurons enhanced the 40 Hz ASSR in the MGB and AC

**Figure 6.**
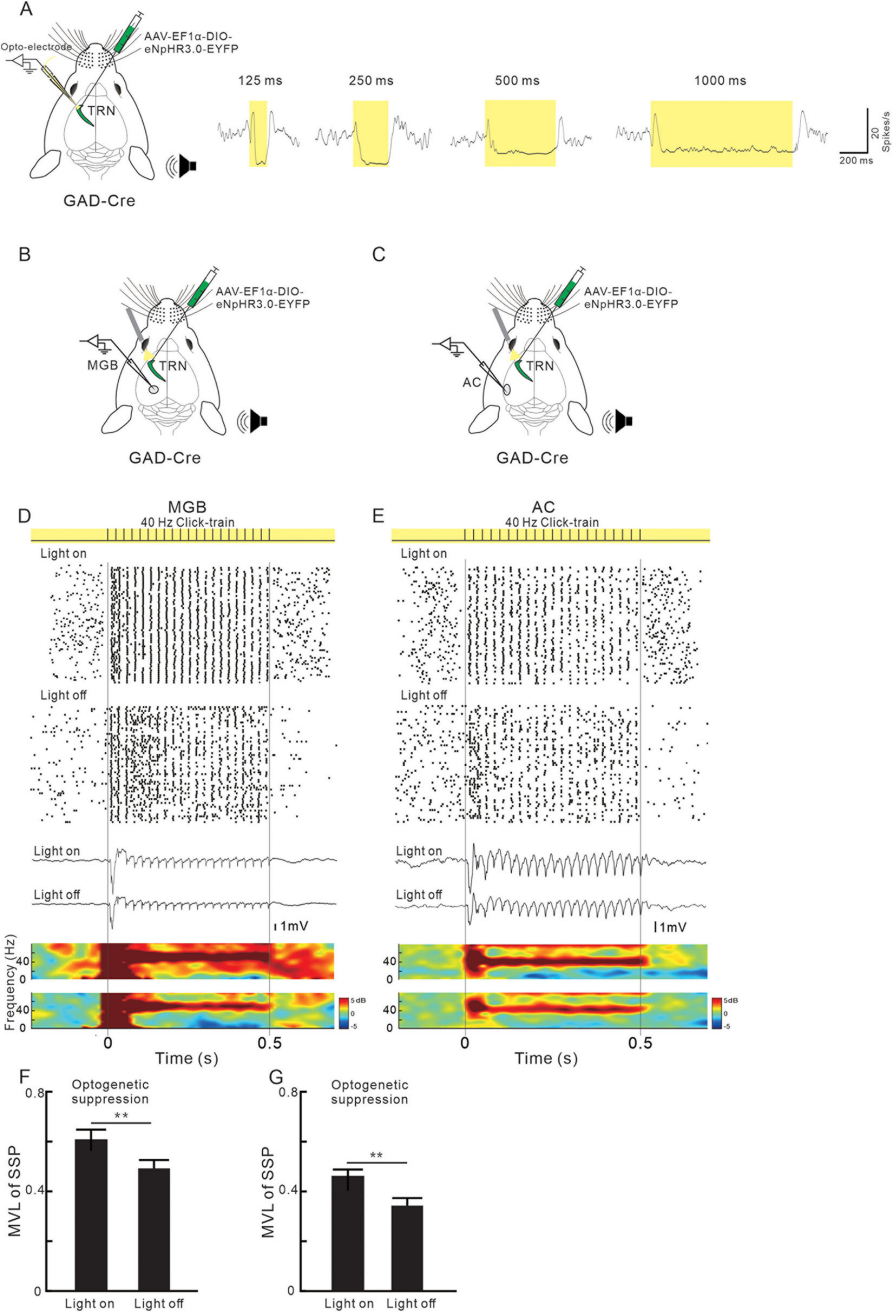
Effect of optogenetic suppression of TRN neurons on sound-evoked responses in the MGB and AC. ***A***, Profiles of TRN SUA in response to yellow light stimuli with different durations. ***B***, ***C***, Schematic diagram of optogenetic suppression of TRN and recording in the MGB and AC, respectively. ***D***, ***E***, SUA and LFP, in the MGB or AC, respectively, in response to 40 Hz sound stimulation with or without yellow light stimulation on the TRN. ***F***, ***G***, Comparison of MVL between the conditions with and without photostimulation (*n* = 31 units from 5 mice; *p < 0.05; paired Student's *t* tests).

Subsequently, we investigated the impact of TRN suppression on the ASSRs in the MGBv and AC. To suppress TRN activity, we utilized yellow photostimulation on GABAergic neurons of GAD-Cre mice, which had expressed eNpHR3.0 by injection of AAV2/9-F1α-DIO-eNpHR3.0-EYFP virus. The effectiveness of photoinhibition was confirmed through an in vivo experiment using an optoelectrode, which demonstrated complete suppression of TRN neuron discharge during the light stimulation period if its duration was longer than 125 ms (Fig. 6*A*). We applied a continuous yellow light stimulation on the TRN for 1 s, completely covering the duration of the 0.5 s sound stimulation (Fig. 6*B–E*). The results revealed that the ability of SUA and LFP to synchronize with the 40 Hz click-train was increased by optogenetic suppression of TRN (Fig. 6*F,G*; *p* < 0.05; paired Student's *t* tests).

### Effects of TRN activation on the behavioral performance of sound discrimination

**Figure 7.**
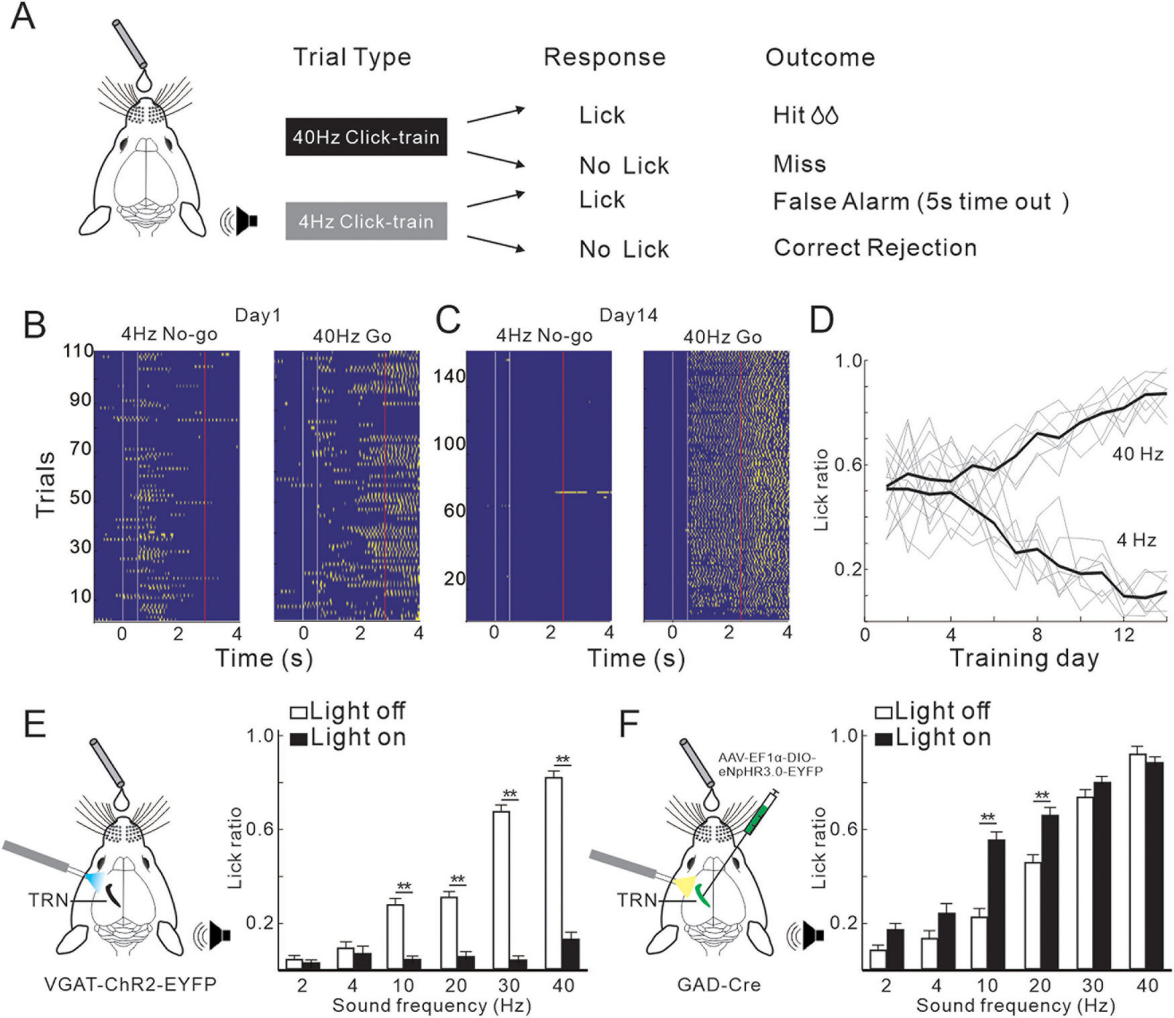
Effects of optogenetic manipulation of TRN on the behavior of mouse performing a sound discrimination task. ***A***, Behavioral paradigm for the Go/No-go task and summary of trial types and outcomes. ***B***, ***C***, Example behavioral sessions at the start and end of training, respectively. Yellow dot, lick; white line, onset and offset of sound presentation; red line, time of reward. ***D***, Function of lick ratio against training day. Gray, result of individual mouse; black, averaged across the mice. ***E***, Lick ratio in response to 6 sounds with different frequencies, which were randomly present in one session. Black and white bar, with and without TRN activation (*n* = 5 mice; **p < 0.01; paired Student's *t* tests). ***F***, Lick ratio with and without TRN suppression (*n* = 4 mice; **p < 0.01; paired Student's *t* tests).

Above results suggest that the activation of TRN has a modulatory effect on the 40 Hz sound-evoked responses in the MGB and AC. In light of this, we sought to investigate how TRN activation influences the behavioral performance of auditory perception. To this end, we trained head-fixed mice on a Go/No-go auditory discrimination task (as outlined in Fig. 7*A*, Materials and Methods, Experimental design) to report the presence of a target or non-target sound (40 vs 4 Hz click-train). With about 2 weeks of training, the mice demonstrated high levels of task proficiency, as evidenced by a marked increase in licking probability at the end of the target sound (Fig. 7*B–D*). After the mice had been well trained to distinguish 40 Hz from 4 Hz click-train (correct rate maintained at level higher than 0.75 for five successive sessions), we then examined their ability to discriminate the sound rhythm by randomly presenting a series of click-train with five different frequencies (2, 4, 10, 20, 30, and 40 Hz) in one test session. The mice showed a higher ratio of lick response to a ≥30 Hz sound and a lower ratio to a ≤20 Hz sound (Fig. 7*E*, white bar). This suggests a psychophysical boundary between 20 and 30 Hz; that means the mice can discriminate a 40 Hz sound from those lower than 20 Hz but cannot from those between 30 and 40 Hz. When TRN was optogenetically activated, the lick rate for ≥30 Hz sounds dramatically decreased (Fig. 7*E*, black bar), indicating that the mouse's ability to detect a high frequency sound was disrupted. On the other hand, when TRN was suppressed, the lick ratio for 10 and 20 Hz was significantly elevated (Fig. 7*F*, black bar), suggesting that the mice treated more lower frequency sounds as 40 Hz sound. Therefore, both activation and suppression of TRN have a significant impact on auditory discrimination behavior.

## Discussion

Despite the widespread application of the 40 Hz ASSR as a translational electroencephalographic biomarker for neuropsychiatric disorders, the generation and regulatory mechanisms underlying the ASSR have not been fully characterized. In this study, we investigated the propagation of rhythmic acoustic stimulation throughout different stages of the auditory thalamocortical hierarchy. We discovered an obvious laminar profile of the 40 Hz ASSR in the AC, with the strongest response occurring in the G layer. Putative GABAergic neurons in the G layer exhibited a higher ability to synchronize with 40 Hz sound stimuli than putative non-GABAergic neurons. Within the MGB, the strongest 40 Hz ASSR of LFP and SUA was observed in the MGBv, which provides the main thalamic inputs to the G layer of the AC. Optogenetic activation of GABAergic neurons in the G layer of AC or the neural projections from the MGBv to the G layer could induce periodic oscillations of LFP in the AC surface, while optogenetic suppression of these neurons reduced the ASSR evoked by 40 Hz sound stimuli. We also discovered that TRN neurons were consistently activated by the 40 Hz sound stimuli but did not synchronize with them. Optogenetic activation or suppression of TRN neurons caused a change in the 40 Hz ASSR in the MGBv and AC. Behavioral experiments further confirmed that both activating and suppressing TRN had an adverse effect on the ability to discriminate 40 Hz sounds. Our results reveal the detailed roles of the TRN–MGB–AC circuit in the generation and regulation of the ASSR, which may aid in the interpretation of clinical ASSR examination results and analysis of the potential neurophysiological mechanisms underlying psychological disorders.

### Propagation of 40 Hz rhythmic activity throughout different stages of the auditory thalamocortical hierarchy

The AC is characterized by its thick, dense, granular layer, which primarily receives thalamocortical projection from the first-order (or lemniscal) auditory thalamus. In a previous study (Li et al., 2021), we reported a laminar-specific feature of the 40 Hz click-train-evoked response in the AC, wherein the strongest amplitude of the evoked neural response was observed in the G layer. Our present study confirms this finding and further identifies that GABAergic neurons in the G layer exhibit a higher synchronization ability to the 40 Hz click-train stimuli. Previous research has proposed that the generation of gamma rhythms is dependent on the precise spiking of inhibitory interneurons, which can control spike timing with great accuracy (Dumont et al., 2016; Adesnik, 2018; Li et al., 2021). Our optogenetic experiments support the pivotal role of GABAergic neurons in the generation of the 40 Hz ASSR in the AC. A recent study on V1 using visual flicker stimuli also found that fast-spiking interneurons in the input layer of V1 showed the strongest phase locking to the gamma-frequency flicker stimuli and phase locking was very weak in L2/3 excitatory neurons (Schneider et al., 2023). However, in this study, we could not differentiate the subtypes of GABAergic interneurons, such as parvalbumin-expressing (PV) and somatostatin-expressing (SST) interneurons. Both of them are proposed to contribute to the generation of gamma oscillation (Hajos and Paulsen, 2009; Ridker et al., 2017; Li et al., 2021). The detailed functions of PV and SST interneurons in the dynamics of 40 Hz ASSR remain unclear. Considering that PV neurons are more frequently located in the G layers, processing large, fast synaptic conductance along with short membrane time constants, they might be the primary contributor to the ASSR observed in the G layer.

The capacity to synchronize with 40 Hz stimuli exhibited variations across the different divisions of MGB. Synchronizing responses were most robust in MGBv and relatively weaker in MGBd and MGBm. These distinct response patterns are consistent with the traditional understanding of MGB's functional anatomy, which entails two distinct thalamocortical projection systems. The lemniscal projection system primarily originates in MGBv, accurately conveys stimulus-specific information, and targets the input layer of AC. This system demonstrates strong frequency tuning and mapping (tonotopic organization). Conversely, the nonlemniscal projection system predominates in MGBd and MGBm. This system targets supra- and infragranular layers of AC and displays broader frequency tuning with little, if any, tonotopic organization (P. Smith et al., 2012; Vasquez-Lopez et al., 2017; Lakatos et al., 2020). In line with this, our results indicate that the rhythmic information of 40 Hz sound was mostly transmitted from MGBv to the G layer of AC. Visual experiments in mice also suggested that gamma synchronization of the lateral geniculate nucleus predominantly drives the input layer of V1 (Schneider et al., 2023). Several studies on the somatosensory cortex have shown that both interneurons and pyramidal neurons in the thalamorecipient layers receive direct thalamic inputs, but the interneurons have a shorter latency of response comparing to the pyramidal neurons (Bruno and Simons, 2002; Cruikshank et al., 2007). Thus, the 40 Hz ASSR recorded in the cortical surface may reflect the function of thalamocortical afferents from MGBv to the GABAergic interneurons in the G layer of AC.

### Regulation of ASSR by the TRN

Various neurological and psychiatric disorders, including SZ, have been associated with abnormal thalamocortical dynamics (Guillery and Sherman, 2002; Llinas et al., 2005; Pratt and Morris, 2015). Therefore, understanding how thalamic relay systems modulate the temporal dynamics of cortical activity has significant translational implications. An effective approach to uncover the role of subcortical systems in temporal modulations of cortical activity is through a combination of optogenetic stimulation and electrophysiological recording. Our study focused on the TRN, an important component of the corticothalamic loop, which plays a vital role in cortical activation, arousal, and attention processing (Halassa et al., 2014, 2015; Vantomme et al., 2019). In rodents, very few GABAergic interneurons are present in the thalamus, and all cortical inputs and outputs between the thalamus pass through the TRN (Halassa and Acsady, 2016). Therefore, understanding the inhibitory mechanisms exerted on the thalamus from the TRN and its ability to control the response of thalamic relay cells and regulate cortical activity is of particular interest. Previous studies have suggested that TRN activation is linked to the generation of cortical spindle oscillations (7–15 Hz; Domich et al., 1986; Halassa et al., 2011; Halassa et al., 2014). Single optogenetic stimulus delivered to the TRN could evoke burst firing in the TRN neurons and spindle oscillations in the cortex (Halassa et al., 2011; Thankachan et al., 2019). During wakefulness, TRN neurons continuously discharge and gate sensory transmission to the cortex (Pinault and Deschenes, 1992). In this study, we found that 40 Hz click-train sounds elicited a series of continuous discharges in the TRN neurons, which did not synchronize with the stimuli. Additionally, our optogenetic results showed that continuous high-frequency optical stimulation could drive TRN neurons to discharge in a mimicking pattern. Furthermore, continuous activation of TRN neurons significantly suppressed the 40 Hz ASSRs in the MGB and AC, whereas optogenetic suppression of TRN activation enhanced their synchronizing responses. In our previous pharmacological study, we found that blocking the glutamatergic activity in the MGB by microinjection of NMDAr antagonists caused a significant decrease in the ASSR in the AC (X. Wang et al., 2020). This result suggests that the ASSR of AC originates from the glutamatergic activity in the MGB. In line with this, the optogenetic activation of TRN may increase the inhibition to MGB glutamatergic neurons resulting in a decrease of ASSR in the AC. Recently, an optogenetic study also found that specially activating TRN neurons, which receive projections from the amygdala, drove an increase in the tone-evoked response amplitude in both the MGB and the AC (Aizenberg et al., 2019), and the increasing effect was stronger in the MGB than the AC. However, that study primarily aimed to investigate neural responses to transient sound stimuli, and the duration of the sound stimuli used was only 50 ms, which might elicit burst firing in TRN neurons. In contrast, our study focused on the neural processing of continuous sound stimuli, which is involved in the tonic firing of TRN neurons. Our electrophysiological results were consistent with behavioral experiments, which showed that optogenetic activation and suppression of TRN reduced the performance of mice in discriminating 40 Hz click-train sounds.

This outcome highlights the filtering role of TRN in auditory processing, which prevents noise or irrelevant information from reaching the auditory cortex (Klug et al., 2018; Baez-Nieto et al., 2022). Both excess and deficiency of the filtering function may disrupt the auditory processing, decreasing the capacity of sound discrimination. Furthermore, the over-activation or suppression of TRN may also cause a change of arousal or attention state, which has a potential effect on the cognitive behaviors (Iidaka et al. 2019). Therefore, appropriate activation of TRN is essential to maintain a suitable gating effect on the auditory processing for repetitive stimuli.

Because a number of studies have reported a reduction of 40 Hz ASSR in individuals with SZ (Light et al., 2006; Krishnan et al., 2009), ASSR deficiency may constitute a premorbid risk marker of SZ. Our electrophysiological results in mice indicate that over-activation of TRN neurons is an important contributor to the reduction of ASSR in the MGB and AC. TRN dysfunction has been suggested to be a key factor in the etiology of SZ (Pratt and Morris, 2015). However, to date there is little information on possible neuropathological changes in the TRN of SZ patients. Because the TRN is too narrow to be resolved by in vivo imaging methodologies such as PET or fMRI, future studies should address this issue by directly conducting the electrophysiological recording and optogenetic manipulation experiments on the TRN neurons of SZ animal models. This approach will enable us to examine whether there is a causal link between TRN dysfunction and ASSR abnormality in the SZ.
