# Supplementary figures and images for: The Thalamocortical Mechanism Underlying the Generation and Regulation of the Auditory Steady-State Responses in Awake Mice
Source: J Neurosci. 2024 Jan 3;44(1):e1166232023. doi: 10.1523/JNEUROSCI.1166-23.2023 (PMC10851679; doi:10.1523/JNEUROSCI.1166-23.2023)

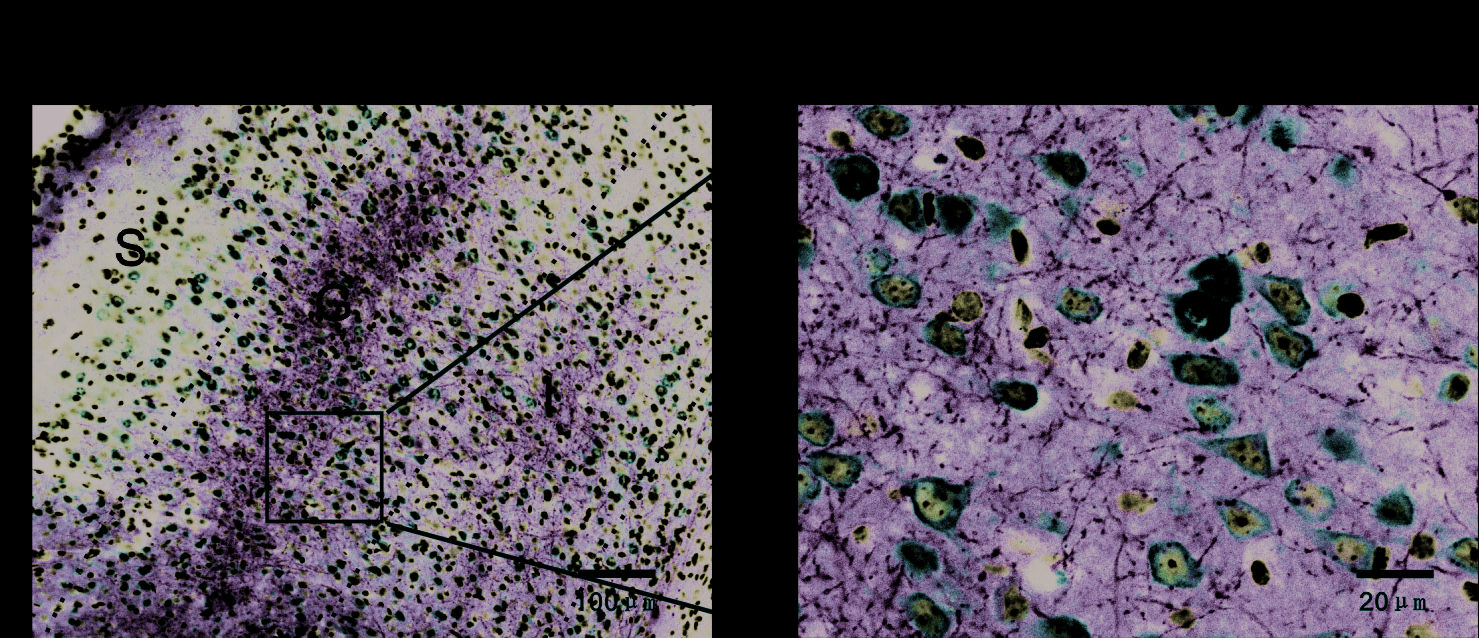

Supplement: Figure 4-1 — (A-B) Laser confocal photographs of DAPI/EYFP/NeuN in the G layer of AC. Blue fluorescence represents nuclear staining (DAPI), green fluorescence represents axonal fibers originating from ChR2-EYFP-expressing projection neurons in the MGBv and red represents NeuN staining. The scale bar in (A) represents 100 μm, 20 μm in (D). Download Figure 4-1, TIF file. [file jneuro-44-e1166232023-s001.tif]
